# Generation, repair and replication of guanine oxidation products

**DOI:** 10.1186/s41021-017-0081-0

**Published:** 2017-08-01

**Authors:** Katsuhito Kino, Masayo Hirao-Suzuki, Masayuki Morikawa, Akane Sakaga, Hiroshi Miyazawa

**Affiliations:** 1 Laboratory of Molecular Biology, Kagawa School of Pharmaceutical Sciences, Tokushima Bunri University, 1314-1, Shido, 769-2193, Sanuki, Kagawa Japan; 2 Laboratory of Xenobiotic Metabolism and Environmental Toxicology, Faculty of Pharmaceutical Sciences, Hiroshima International University (HIU), 5-1-1 Hiro-koshingai, Kure, 737-0112, Hiroshima, Japan; 3 Laboratory of Medicinal Chemistry, Kagawa School of Pharmaceutical Sciences, Tokushima Bunri University, 1314-1, Shido, 769-2193, Sanuki, Kagawa Japan

**Keywords:** Oxidation, Guanine, G:C-C:G transversions, 2,2,4-triamino-5(2*H*)-oxazolone (oz), Base pair, DNA polymerase, Repair, Contiguous oz, Highest occupied molecular orbital (HOMO), Quadruplex DNA

## Abstract

Guanine is the most readily oxidized of the four DNA bases, and guanine oxidation products cause G:C-T:A and G:C-C:G transversions through DNA replication. 8-Oxo-7,8-dihydroguanine (8-oxoG) causes G:C-T:A transversions but not G:C-C:G transversions, and is more readily oxidized than guanine. This review covers four major findings. (i) 2,2,4-Triamino-5(2*H*)-oxazolone (Oz) is produced from guanine and 8-oxoG under various oxidative conditions. Guanine is incorporated opposite Oz by DNA polymerases, except REV1. (ii) Several enzymes exhibit incision activity towards Oz. (iii) Since the redox potential of GG is lower than that of G, contiguous GG sequences are more readily oxidized by a one-electron oxidant than a single guanine, and OzOz is produced from GG in double-stranded DNA. Unlike most DNA polymerases, DNA polymerase ζ efficiently extends the primer up to full-length across OzOz. (iv) In quadruplex DNA, 3′-guanine is mainly damaged by one-electron oxidation in quadruplex DNA, and this damage depends on the highest occupied molecular orbital (HOMO). The oxidation products in quadruplex DNA are different from those in single-stranded or double-stranded DNA.

## Background

Cellular DNA is constantly oxidized by various endogenous and exogenous agents, and the resulting DNA damage may increase the risk of developing cancer and other diseases [1]. Guanine has the lowest redox potential of the four DNA bases [2] and is therefore the most easily oxidized.

During DNA replication, adenine incorporation opposite a guanine oxidation product induces a G:C-T:A transversion, whereas guanine incorporation opposite a guanine oxidation product causes a G:C-C:G transversion. These mutations (see references in [3]) are found in many important genes, and in particular at CpG sites in the *p53* tumor suppressor gene and in codons 12 and 13 of the *K-ras* oncogene [4–6]. Therefore, it is important to analyze nucleotide incorporation and DNA extension for each guanine oxidation product in order to elucidate the mechanisms underlying the generation of G:C-T:A and G:C-C:G transversions.

**Fig. 1:**
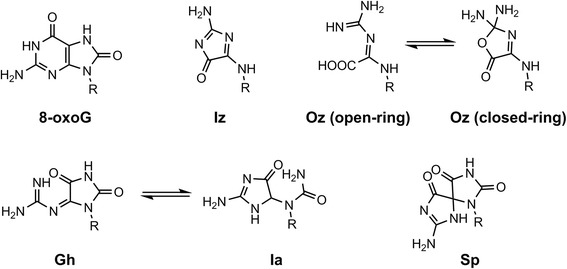
Oxidation products of guanine

**Fig. 2:**
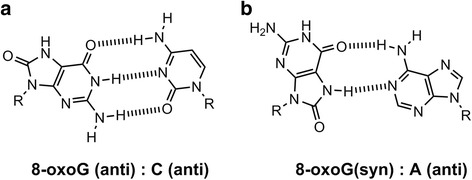
The structures of (**a**) 8-oxoG (*anti*):C (*anti*) and (**b**) 8-oxoG (*syn*):A (*anti*) [8–10]

8-Oxo-7,8-dihydroguanine (8-oxoG) (Fig. 1) is one of the most common oxidative DNA lesions and is formed under various oxidative conditions. 8-OxoG has been studied extensively, has significant biological impact, and is a ubiquitous marker of oxidatively damaged DNA [1, 7]. Structural analyses have revealed that 8-oxoG adopts either an *anti* or *syn* conformation: 8-oxoG in the *anti* conformation forms Watson-Crick base pairs with cytosine, while the lesion in the *syn* conformation utilizes the Hoogsteen edge of the lesion to form a base pair with adenine [8–10] (Fig. 2). Furthermore, DNA polymerases incorporate adenine in addition to cytosine opposite 8-oxoG [7, 11–19], and 8-oxoG induces a G:C-T:A transversion in *Escherichia coli* (*E. coli*) [20, 21] and mammalian cells [22–24].

Although 8-oxoG causes G:C-T:A transversions, the mechanism underlying the generation of G:C-C:G transversions cannot be explained by 8-oxoG. In addition, 8-oxoG is more readily oxidized than guanine because of its lower oxidation potential [25, 26], and thus various oxidative lesions are produced by the oxidation of 8-oxoG. Therefore, it is necessary to study both 8-oxoG and other oxidized guanine lesions in order to understand the various phenomena caused by guanine oxidation.

### Guanine oxidation products causing G:C-C:G transversions form base pairs by hydrogen bonding with guanine

As previously mentioned, G:C-C:G transversion is caused by incorporation of guanine opposite an guanine oxidation product. In this section we first describe the “A-rule”. Adenine is the most common base incorporated opposite an abasic site by DNA polymerases [27], suggesting that, of the four natural bases, adenine forms the most hydrophobic interactions and exhibits the best spatial compatibility with an abasic site. Adenine incorporation may not necessarily require the formation of hydrogen bonds with various damaged DNA sites in template DNA. In contrast, the preferential insertion of guanine requires hydrogen bond formation [12], in addition to hydrophobic interactions and spatial compatibility, with the templating base. Thus, in order to reveal the lesions causing G:C-C:G transversions, it is important to investigate guanine oxidation products that form base pairs by hydrogen bonding with guanine.

### Potential guanine oxidation products causing G:C-C:G transersions

**Fig. 3:**
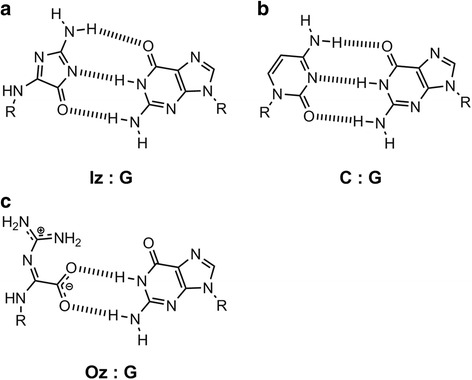
The structures of (**a**) proposed Iz:G [30, 34], (**b**) C:G and (**c**) proposed Oz:G base pairs [12, 42]

2,5-Diamino-4*H*-imidazol-4-one (Iz) is an oxidized product of both guanine and 8-oxoG [28–31] (Fig. 1), in both single- and double-stranded DNA [32, 33]. We previously proposed that the H-bonding donor and acceptor abilities of Iz are similar to those of cytosine [30, 34] (Fig. 3a), and reported that the calculated stabilization energy of the Iz:G base pair is similar to that of the C:G base pair [34] (Fig. 3b). Melting temperature measurements revealed that the Iz:G base pair is more stable than Iz:A, Iz:C, and Iz:T [35]. Moreover, previous reports indicated that Iz mainly causes G:C-C:G transversions in vitro and in vivo [34, 36].

Iz is slowly hydrolyzed to 2,2,4-triamino-5(2*H*)-oxazolone (Oz) (Fig. 1) in neutral aqueous solution, and its half-life is 147 min under physiological conditions [28]. Additionally, 2–6 molecules of Oz are detected per 10^7^ guanine bases in liver DNA [37], and it was recently shown that the amount of Oz is significantly increased in the presence of 5-methylcytosine [38]. Thus, the biological influence of Oz in DNA replication is likely larger than that of Iz.

Klenow fragment exo^−^ incorporates adenine opposite Oz in vitro [39], and Oz causes G:C-T:A transversions in *E. coli* [20]. In contrast, in our in vitro reaction system, Klenow fragment exo^−^ incorporates either adenine or guanine opposite Oz [12]. Moreover, we revealed that DNA polymerases α, β, δ, and ε each incorporate only guanine opposite Oz; DNA polymerases γ, κ, and *Sulfolobus solfataricus* DNA polymerase IV, in addition to Klenow fragment exo^−^, each incorporate guanine and adenine; DNA polymerase η incorporates guanine, adenine and cytosine; DNA polymerases ζ and ι each incorporate guanine, adenine, cytosine and thymine; and REV1 incorporates cytosine [12, 40, 41]. These analyses indicate that DNA polymerases, except REV1, incorporate guanine opposite Oz [12, 40, 41]. We predicted that Oz can form a stable base pair with guanine, and that this Oz:G base pair has two hydrogen bonds and is planar (Fig. 3c) [12, 42]. We recently performed thermal denaturation experiments and reported that the Oz:G base pair is more thermodynamically stable than Oz:A, Oz:C, and Oz:T [43].

In addition, DNA polymerases α, β, γ, δ, ε, η, κ, and ζ each elongate the primer to full length across Oz [12, 40, 41]. Thus, Oz appears to be an oxidized lesion related to the induction of G:C-C:G transversions. In particular, DNA polymerase ζ elongates beyond Oz with almost the same efficiency as beyond G [40], but not beyond tetrahydrofuran (THF; a chemically stable abasic site analog), 8-oxoG, or *O* ^6^-methylguanine [18, 44]. These data indicate that DNA polymerase ζ is an effective error-prone replication polymerase for Oz. Moreover, only REV1 incorporates cytosine opposite Oz, and the frequency of cytosine insertion follows the order Oz > guanidinohydantoin (Gh) > THF > 8-oxoG [40]. Given that REV1 can interact with DNA polymerases [45–49], the collective results to date suggest that REV1 together with DNA polymerase ζ might prevent G:C-C:G transversions [40]. Such systems for preventing mutation are seen in other DNA polymerases [50, 51].

Recently, other groups reported that Gh and spiroiminodihydantoin (Sp), which are oxidation products of 8-oxoG (Fig. 1) [29, 52, 53], induce G:C-T:A and G:C-C:G transversions [12, 21, 41, 54–57]. Gh can isomerize to iminoallantoin (Ia) (Fig. 1) [29]. The efficiency of guanine incorporation opposite Gh/Ia is higher than that opposite Sp [54], and guanine insertion opposite Oz is more efficient compared to that opposite Gh/Ia [12]. In addition to guanine incorporation, extension beyond Oz is more efficient than that beyond Gh/Ia or Sp [12, 41, 54]. This phenomenon is believed to depend on the stacking effect and structural distortion [51, 58], in addition to the stability of the base pair [42]: Oz has no *sp* ^*3*^ carbon and is planar, whereas Gh/Ia and Sp are nonplanar due to their *sp* ^*3*^ carbon [59]. Thus, we believe that Oz is a more important cause of G:C-C:G transversions than Gh/Ia or Sp.

### Reaction of oligonucleotides containing oz with repair enzymes

Cells utilize a number of mechanisms to prevent mutagenic effects of the above-described types of DNA damage, and have various enzymes that repair DNA damage such as 8-oxoG [7, 60, 61].

*E. coli* formamidopyrimidine DNA glycosylase and endonucleases III enzymes excise Oz from double-stranded DNA oligomers [39, 62]. In addition, human NTH1 and NEIL1, which are homologues of *E. coli* endonucleases III and VIII, remove Oz as efficiently as they remove 5-hydroxyuracil, and their reactivity towards Oz is higher than towards 8-oxoG [63]. Since Oz is more sensitive than 8-oxoG to treatment with piperidine, this difference in reactivity depends on the strength of the *N*-glycosidic bond [63].

We analyzed the incision activities of other repair enzymes on Oz. Chlorella virus pyrimidine dimer glycosylase, which exhibits incision activity towards cyclobutane pyrimidine dimer, reacts with Oz-containing double-stranded DNA. Its reactivity towards Oz is higher than towards 8-oxoG, indicating that reactivity is dependent on *N*-glycosidic bond strength, similar to human NEIL1 and NTH1 [64]. However, this repair enzyme exhibits moderate activity towards Oz compared to its activity towards cyclobutane pyrimidine dimer [64].

Endonuclease IV, an apurinic/apyrimidinic endonuclease, incised Oz with an observed activity one-third to one-fourth of that towards THF [64], but with greater efficiency than its activity towards Gh. These results suggest that Oz may be more structurally similar to an abasic site than is Gh [64].

Endonuclease V, which exhibits activity towards hypoxanthine residues, is also active towards Oz. This activity is lower than that towards hypoxanthine at high concentrations of endonuclease V, but endonuclease V recognizes Oz much more efficiently than it does Gh. Endonuclease V can recognize uracil but not thymine, suggesting that the 5′-methyl group is critical for recognition by endonuclease V [65]. As described previously [64], unlike the closed-ring structure of Oz (Fig. 1), Gh has a moiety protruding from the ring, similar to thymine. Thus, endonuclease V can recognize Oz but not Gh.

Human OGG1 and AP endonuclease 1 cannot excise Oz residues [63, 66], and single-strand-selective monofunctional uracil-DNA glycosylase 1 exhibits no incision activity towards Oz (data not shown). *E. coli* Mut Y cannot act on Oz:G and Oz:A lesions (data not shown). Oz is a weak substrate for human nucleotide excision repair because the damaged site is less bulky than that of pyrimidine (6–4) pyrimidone photoproducts, making it difficult for XPC-RAD23B to recognize Oz [67].

The data available to date indicate that human NEIL1 and NTH1 are the most likely repair enzymes for Oz. However, human NEIL1, NTH1, *E. coli* endonucleases III, and formamidopyrimidine DNA glycosylase can all excise Oz from double-stranded DNA, regardless of the type of base opposite Oz [39, 62, 63]. If the base opposite Oz is adenine, guanine, or thymine before base excision repair, genetic information is altered in subsequent replications. Thus, there may be unknown enzymes which can remove Oz accurately from oligonucleotides in which Oz is paired to a C, just as human OGG1 can remove 8-oxoG. Alternatively, as mentioned earlier [40], REV1-DNA polymerase ζ may prevent G:C-C:G transversions.

### Does OzOz obstruct DNA synthesis by DNA polymerases?

Contiguous guanines (GG), which exist in many important genomic regions such as the *K-ras* oncogene [7], are more readily oxidized than a single guanine due to their lower redox potential [68–72]. The oxidation of GG sequences under high oxidation conditions results in a contiguous oxidized guanine lesion. We previously reported that IzIz is produced by the oxidation of GG in single- and double-stranded DNA [30], suggesting that hydrolysis of these Iz molecules produces two contiguous Oz molecules (OzOz). It is expected that OzOz stalls DNA synthesis more effectively than a single Oz and thus represents more serious DNA damage than a single Oz.

Our analysis showed that DNA polymerase κ did not incorporate any nucleotide opposite OzOz [73]. Klenow fragment exo^−^ preferentially incorporated one adenine opposite the 3′ Oz of OzOz lesions, REV1 incorporated cytosine, and DNA polymerase β incorporated guanine [73]. DNA polymerase α incorporated guanine, adenine and cytosine opposite the 3′ Oz of OzOz lesions, and guanine was incorporated more readily than adenine and cytosine [73]. DNA polymerase ι slightly incorporated guanine, adenine and thymine [73]. Whether a base is incorporated or not, these polymerases cannot elongate the primer up to full-length across OzOz [73].

In contrast, DNA polymerase η elongated the primer up to full-length across OzOz lesions with modest efficiency compared to across cyclobutane pyrimidine dimer lesions, with the synthesis of most DNA strands stalling at the 3′ or 5′ Oz of OzOz [73]. In contrast, DNA polymerase ζ could efficiently elongate the primer up to full-length across OzOz [73], suggesting that DNA polymerase ζ is an important enzyme for translesion synthesis past both single and contiguous Oz molecules. In addition, DNA polymerase ζ incorporates all nucleotides opposite both the 3′ and 5′ Oz [73] and is an error-prone DNA polymerase for OzOz.

### Photooxidation in quadruplex DNA

**Fig. 4:**
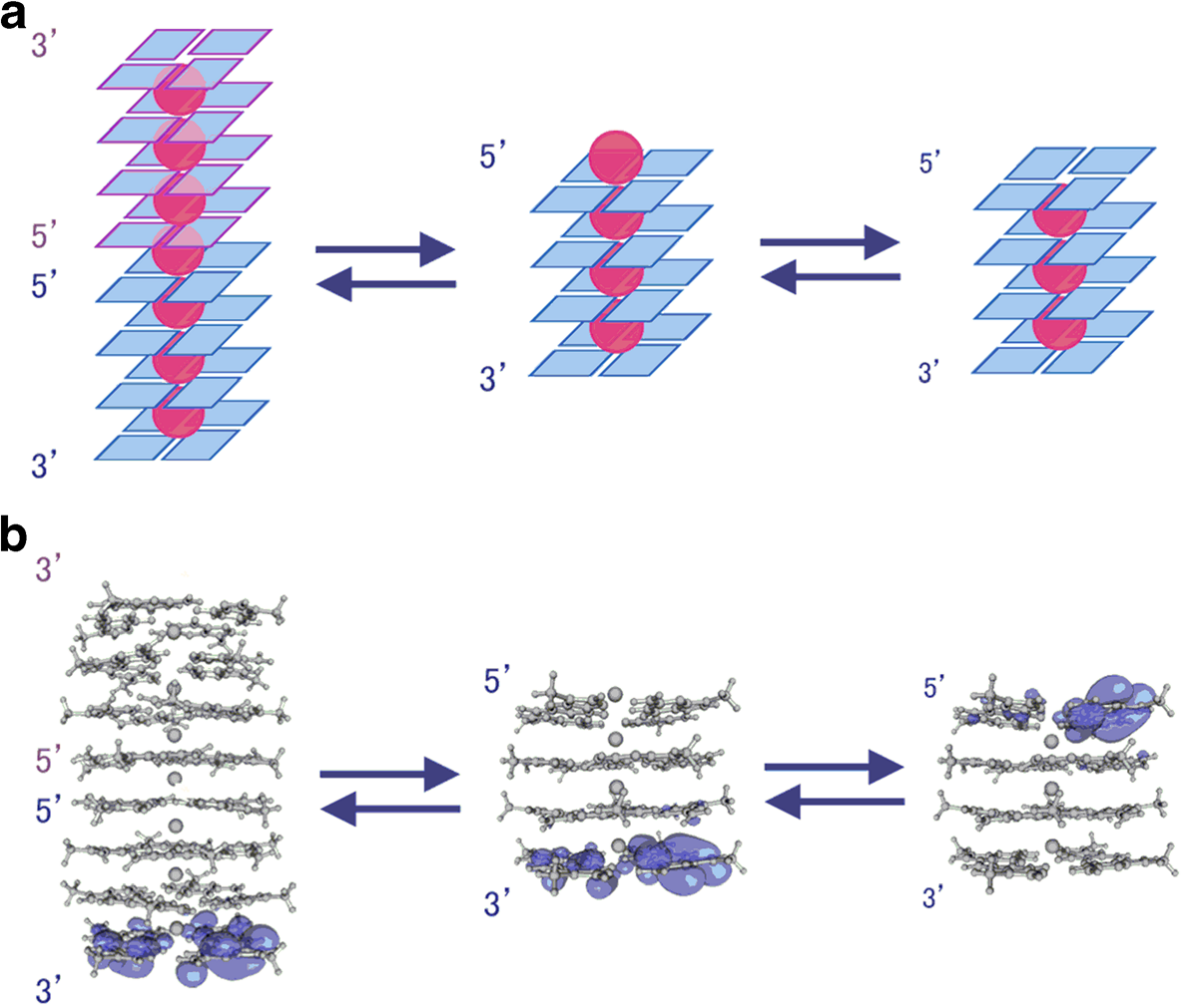
The proposed equilibrium and HOMO of d(TGGGGT)_4_. **a** Models of stacked G-quadruplexes. The blue planes are guanine, and the red balls are K^+^. **b** The localization of the HOMO. HOMOs were calculated at the B3LYP/6-31G* levels [32, 77, 79]. These figures are adapted from data reported previously

Guanine-rich sequences such as telomeres can fold into quadruplex structures [74–76]. In double-stranded DNA, the one-electron oxidation of guanine in contiguous guanine sequences is dependent on the localization of the highest occupied molecular orbital (HOMO) [33, 68, 69]. Unlike double-stranded DNA [33], the 3′-guanine of d(TGGGGT) is mainly oxidized in quadruplex DNA, and the estimated HOMO is localized on the 3′-guanine [32] (Fig. 4b). Given our current understanding of double-stranded DNA [33, 68, 69] and quadruplex DNA [32], the selective one-electron oxidation of guanine occurs at the localized HOMO regardless of the DNA structure.

In particular, the 5′-capping cation (d(TGGGGT)_4_ + 4 K^+^) is important for HOMO localization on the 3′-guanine [77] (Fig. 4), and two quadruplexes may stack at their 5′ ends [78] (d(TGGGGT)_8_ + 7 K^+^ in Fig. 4a) in water because the HOMO of this structure is localized on the 3′-guanine [79] (Fig. 4b).

On the other hand, the guanine oxidation products depend on the DNA structure. For example, Iz is a major product in single-stranded DNA, whereas 8-oxoG and dehydroguanidinohydantoin (Ghox) are mainly formed in quadruplex DNA [32]. Iz, 8-oxoG, Ghox and Gh are formed in double-stranded DNA, and are also major oxidation products found in single-stranded and quadruplex DNA [33].

**Fig. 5:**
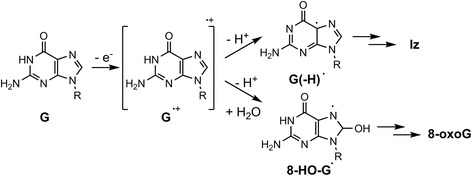
Two pathways for the oxidation of guanine

**Fig. 6:**
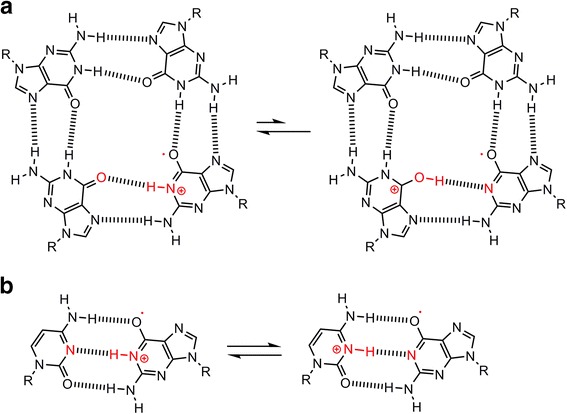
Proton shifts in (**a**) the G^•+^:G base pair of quadruplex DNA and in (**b**) the G^•+^:C base pair of double-stranded DNA

The mechanism of guanine oxidation in single-stranded, double-stranded, and quadruplex DNA may be explained as follows. One-electron oxidation of guanine generates a guanine radical cation (G^•+^) [80], followed by two degradation pathways [32, 33] (Fig. 5). In one pathway, G^•+^ is deprotonated at the N1 position, followed by generation of Iz. In another pathway, G^•+^ is hydrated and deprotonated, followed by the generation of 8-oxoG, Ghox, and Gh. There is a hydrogen bond between the N1 proton of G^•+^ and the O6 of guanine in quadruplex DNA (Fig. 6a), and a hydrogen bond is formed between the N1 proton of G^•+^ and the N3 of cytosine in double-stranded DNA (Fig. 6b). Therefore, deprotonation of G^•+^ is significantly inhibited in quadruplex DNA and partially inhibited in double-stranded DNA. We estimate the ease of deprotonation to follow the order: single-stranded DNA > double-stranded DNA > quadruplex DNA [33], and this order explains the differences in oxidation products in each type of DNA structure.

## Conclusions

Based on the A-rule, adenine incorporation does not necessarily require hydrogen bonds with the guanine oxidation product, whereas we think that guanine incorporation requires the formation of hydrogen bonds. Therefore, to identify candidates causing G:C-C:G transversions, we searched guanine oxidation products forming base pairs that hydrogen bond with guanine. We used several DNA polymerases to determine that Oz can cause G:C-C:G transversions, in addition to Iz, Gh, and Sp. DNA polymerase ζ is a particularly effective error-prone replication polymerase for Oz. Some repair enzymes exhibit incision activity towards Oz, and thus incision of Oz prior to guanine incorporation inhibits mutations.

In double-stranded DNA, contiguous GG is more readily oxidized by a one-electron oxidant than is a single G. OzOz, an oxidation product of GG, obstructs DNA synthesis by most DNA polymerases, and only DNA polymerase ζ acts on OzOz efficiently.

The 6-mer DNA d(TGGGGT) containing contiguous GGGG is the shortest oligomer among the quadruplex-formative sequences in the presence of ions. The 3′-guanine of contiguous GGGG is mainly oxidized, and this is attributed to the HOMO. In addition, guanine oxidation products formed in quadruplex DNA are different from those in other DNA structures, likely due to the ease of deprotonation of the guanine radical cation.

## Abbreviations

8-oxoG: 8-oxo-7,8-dihydroguanine
*E. coli*: *Escherichia coli*
Gh: guanidinohydantoin
Ghox: dehydroguanidinohydantoin
HOMO: highest occupied molecular orbital
Ia: iminoallantoin
Iz: 2,5-Diamino-4*H*-imidazol-4-one
Oz: 2,2,4-triamino-5(2*H*)-oxazolone
Sp: spiroiminodihydantoin
THF: tetrahydrofuran
